# Rush progression and fatal result of septic shock related to central line catheter infection in cirrhosis patient with brain stroke

**DOI:** 10.1186/s12883-018-1166-5

**Published:** 2018-09-29

**Authors:** Nobuhiko Arai, Yutaka Mine, Hiroshi Kagami, Makoto Inaba

**Affiliations:** 1 Department of Neurosurgery, Saiseikai Yokohamashi Tobu hospital, 3-6-1 Shimosueyoshi, Tsurumi-ku, Yokohama, Kanagawa 230-8765 Japan; 2 Department of Endovascular Surgery, Saiseikai Yokohamashi Tobu Hospital, Yokohama, Kanagawa 230-8765 Japan; 3 Department of Physiology, Keio University School of Medicine, Tokyo, 160-8582 Japan; 4 Department of Clinical Research, Tochigi Medical Center, National Hospital Organization, Utsunomiya, Tochigi, 320-8580 Japan

**Keywords:** Catheter related blood stream infection(CRBSI), *Klebsiella pneumoniae*, Subarachnoid hemorrhage (SAH), Endotoxin, Procalucitonin, Stroke, CIDS (central nervous system injury-induced immunodepression syndrome)

## Abstract

**Background:**

Catheter-related blood stream infection (CRBSI) is one of the most common intractable healthcare-associated infections because catheters can be easily contaminated by resistant bacteria, and is associated with a high mortality. Central lines are currently used for administering medication to patients with severe stroke, and may thus cause CRBSI.

**Case presentation:**

A 71-year-old woman with cirrhosis presented with subarachnoid hemorrhage (SAH) that was treated by clipping surgery. On postoperative day (POD) 38, sudden high fever (40.3 °C) was detected; the patient died a few hours later. Blood and central line cultures were positive for *Klebsiella pneumoniae* that may have caused CRBSI and endotoxin shock. In this case, the duration from fever detection to death was notably short. Additionally, inflammatory markers such as white blood cells (WBC) or C-reactive protein (CRP) were almost within normal ranges, even a few hours after fever was detected and before death. Cirrhosis was considered to be the cause of these phenomena.

**Conclusion:**

The timely diagnosis and complete treatment of patients with liver cirrhosis who develop CRBSI are highly challenging. We suggest that clinicians should rigorously apply preventive measures and strengthen CRBSI monitoring, especially in cirrhosis-associated cases.

## Background

Catheter-related blood stream infection (CRBSI) is one of the most common healthcare-associated infections [1]. CRBSI can be intractable because several resistant bacterial strains can cause CRBSI, and is thus associated with high mortality [2]. Therefore, CRBSI prevention, early detection, and therapy are key elements of patient care. Berenholtz et al. showed that appropriate infection preventive measures can completely prevent CRBSI [3]. These measures including appropriate cleaning of hands; inserting the catheter at sites that are more likely to be sterile and avoiding sites such as the femoral vein; the use of sterile gloves, hat, mask, and gowns; application of sterile dressing; and unnecessary catheter removal daily. It must be noted that while complete prevention of CRBSI can be achieved [3], Central lines are currently used for patients with severe stroke for the administration of medication; infections of these catheters may lead to CRBSI. Additionally, stroke patients develop significant immunodepression, characterized by lymphopenia, upregulation of anti-inflammatory cytokines, and splenic atrophy. This immunodepression clearly manifests during the post-stroke period, when the systemic infection rate is high [4].

Herein, we observed a case of severe endotoxin shock induced by CRBSI in a patient stroke suffices, experienced. The patient also had liver cirrhosis induced by hepatitis type B virus. It took only 24 h for him to dies since the first symptom (high fever) of infection was detected. Our case demonstrates the increasingly rapid progression of CRBSI in the presence of liver cirrhosis, as well as the importance of immediate treatment after accompanying diseases or other organ complications are present and the appropriate application of measures to prevent CRBSI.

## Case presentation

**Fig. 1:**
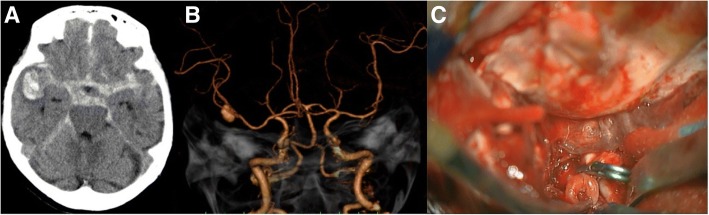
SAH due to right middle cerebral artery aneurysm. Preoperative CT and 3D- CTA and surgical view shows the details of SAH. **a** Preoperative head CT showing diffuse subarachnoid hemorrhage with right temporal hematoma. **b** Preoperative 3D-CTA revealing a right middle cerebral artery aneurysm, which is 12 mm in a diameter. **c** Ruptured right MCA aneurysm was clipped with Sugita titanium clip (arrow). CT: computed tomography, CTA: CT angiography

A 71-year-old woman without any significant medical history presented to the emergency room (ER) of our hospital with slight drowsiness. The hepatitis B surface (HBs) antigen and elevated transaminase levels were detected on a blood examination, revealing chronic hepatitis. The patient contracted the HB virus while receiving transfusion during a cesarean section. Head computed tomography (CT) revealed a subarachnoid hemorrhage (SAH) with a right temporal hematoma and an aneurysm on the right M1-M2 bifurcation (Fig. 1a, b). The SAH was believed to be caused by a right middle cerebral artery (MCA) aneurysm that was classified as World Federation of Neurosurgical Societies Grading of SAH (WFNS) Grade II and CT Fisher Group III. We maintained her systolic blood pressure (SBP) under 120 mmHg and mildly sedated the patient in the ER. Subsequently, a procedure to clip the ruptured aneurysm was performed. After the craniotomy and dural incisions, subdural and massive temporal hematomas with severe brain swelling were detected. Massive bleeding occurred before we could properly reach the aneurysm, implying that the aneurysm reruptured before or during the operation. A temporary clip was quickly set on the right M1 trunk. Following this, a permanent clip was appropriately applied to the aneurysmal neck to close it (Fig. 1c). The removal of the temporary clip took 15 min. One day after surgery, the follow-up CT showed low density in almost the entire right MCA territory, suggesting an infarction. One month after surgery, the patient gradually recovered through rehabilitation, although she had hypoalbuminemia due to malabsorption and cirrhosis.

**Fig. 2:**
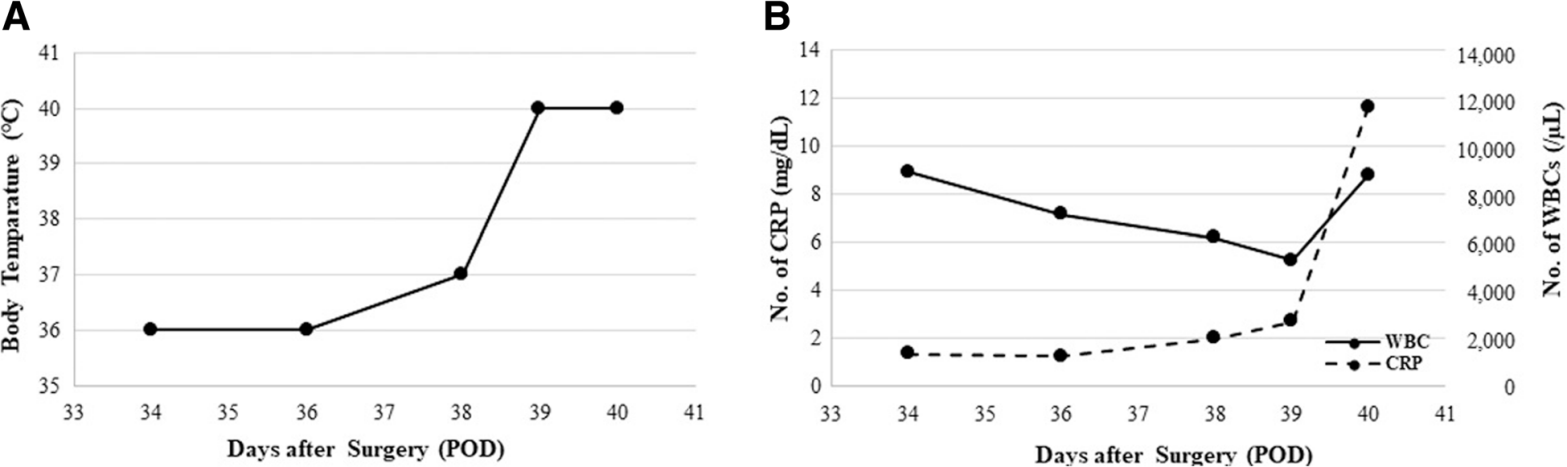
Time course of vital parameters (body temperature, WBC and CRP). These graphs show the time course about body temperature, numbers of WBC and CRP indicating the rush progression on our case. **a** Body temperature. It was raised at the midnight of 38POD. **b** WBC and CRP. They did not show such abnormal readings compared to patients’ condition at the 39POD. Those parameters suddenly increased and the patient died on the 40POD. BP; blood pressure, WBC; white blood cell, CRP; C-reactive protein

**Fig. 3:**
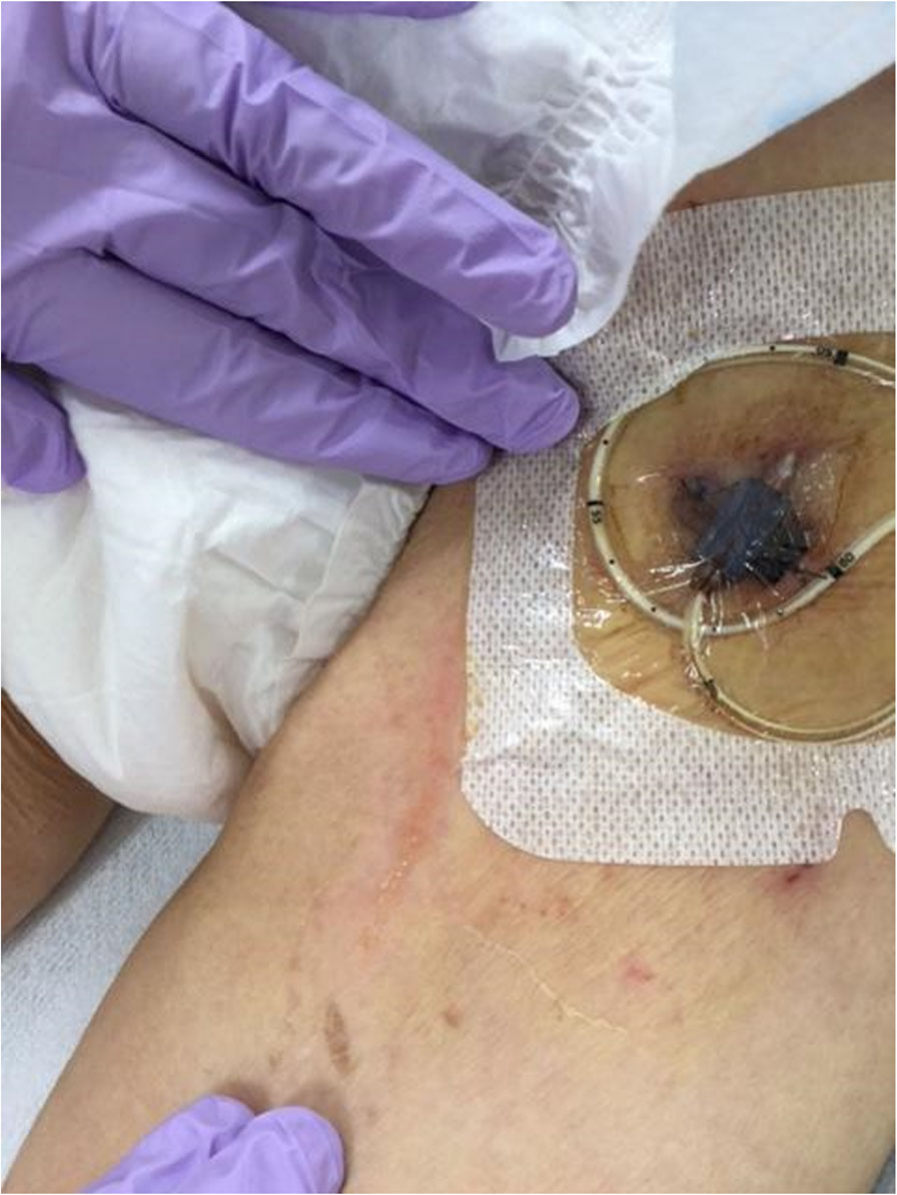
Left femoral vein catheter in 2 weeks might be the origin of infection. Femoral catheter inserted via left femoral vein covered by transparent covering tissue. It was dirty and seemed to be infected, suggesting the origin of infection and CRBSI

On day 38, she suddenly developed high fever (40.3 °C) at midnight. However, the following morning, i.e., on postoperative day (POD) 39 (Fig. 2a), her blood test showed acceptable levels of white blood cell (WBC) and C-reactive protein (CRP) (5250 cells/μL and 2.72 mg/dL, respectively) (Fig. 2b). As her fever reduced, it was believed that the fever was caused by central nervous system (CNS) damage. We immediately completed a general culture workup of the sputum, urine, and blood to understand the origin of the fever. Thus, we intentionally postponed antibiotic administration till the culture workup was completed. However, her SBP gradually declined to 70 mmHg on POD 40. A on-call doctor started vasopressor drugs administration and stabilized her SBP to approximately 100 mmHg. Though WBC and CRP levels were 8810 cells/μL and 6.56 mg/dL, respectively (Fig. 2b), the number of platelets was extremely low (less than 10000/μL) and the fibrin/fibrinogen degradation product (FDP) level was 62.1 μg/mL, suggesting disseminated intravascular coagulation (DIC). Although the spinal tap showed no evidence of meningitis, two sets of blood culture revealed the existence of gram-negative rod bacteria, strongly indicating a septic shock. Although we inserted a central venous catheter (CVC) in a sterile manner, the central line catheter seemed to be contaminated by traces from stools or urine (Fig. 3). Thus, we immediately removed the catheter and started treatment with cefepime, a fourth-generation cephem type antibiotic. Only two hours after antibiotic administration, her SBP reduced dramatically and she became unresponsive to high dose vasopressor (epinephrine). The patient died at 11:00 a.m. on POD 40, one and a half days after fever onset. After her death, the causative agent was confirmed to be *Klebsiella pneumoniae*, as observed by blood culture and catheter analyses. These findings imply that CRBSI might have occurred, inducing a rapid decline in the patient’s condition and resulting in death in a stroke patient with additional organ failure.

## Discussion and conclusions

We present the case of a patient with cirrhosis who had an extremely accelerated disease time course, as death occurred one and a half days after high fever was detected. This shows that CRBSIs can become fatal for patients with cirrhosis rapidly. Additionally, in the present case, liver cirrhosis complicated and delayed the detection of the initial sign of infection as well as the intervention. As CRBSIs accompanied by other organ complications might be fatal, preventative measures against CRBSI should be applied rigorously.

### CRBSI in patients with stroke, CNS injury-induced immunodepression syndrome (CIDS), and cirrhosis-associated immune dysfunction (CAID)

CRBSI is common and can be fatal, especially in patients with accompanying organ failure [2]. Currently, it is not clear whether stroke patients are more susceptible to CRBSI. Some reports have described a few CRBSI cases in the stroke care unit (SCU) [5, 6]. However, those data may not be accurate, and there could have been many false negative cases because blood cultures are not performed for all febrile patients in the SCU, even though they may have a strong risk of developing CRBSI. In contrast, patients with severe stroke receive several medications, such as parental nutrition for massive diarrhea or complicated constipation; antibiotics for aspiration pneumonia, urinary tract infection (UTI) or meningitis; hemodynamic volume therapy for vasospasm following SAH; or antiplatelet therapy for cerebral infarction and vasospasm. Therefore, the use of central line catheters is necessary for these patients, and these should be frequently changed in the SCU and intensive care unit, in order to prevent infection.

Stroke and other CNS injury are known to create a systemic immunosuppression, rendering patients more prone to infections. CIDS is characterized by lymphopenia and immune dysfunctions; in particular, it is characterized by functional deactivation of T-helper (Th) 1 and natural killer (NK) cells, and monocytes/macrophages. Catecholamines have been shown to suppress Th1 activity, promote Th2 cell differentiation, and trigger interleukin (IL)-10 secretion. A correlation between elevated levels of metanephrine and the incidence of post-stroke infections has been demonstrated. Experimental data indicated that an impaired early catecholamine-mediated lymphocyte response, particularly a reduced interferon gamma (IFNγ) secretion by T and NK cells, could be an essential cause of the impaired antibacterial defense after brain ischemia [7]. As a form of post-stroke immunodepression, CIDS could have contributed to the CRBSI observed in the present case.

CAID alters both innate and acquired immunity [8] and results in immunodeficiency as well as systemic inflammation [9]. Endotoxemia could enhance the inflammatory host response and expansive systemic bacterial infection by CAID, resulting in septic shock, multi-organ dysfunction, and death [10–12]. It has also been reported that cirrhosis increases levels of IL-6, tumor necrosis factor alpha (TNF-α), endotoxin, and nitric oxide that induce vasodilatation [13] and reduce C-protein as well as coagulation factor levels [12].

### CAID status and infection marker sensitivity

With a mortality rate of approximately 30% [14], CAID patients have a higher mortality risk than do patients with normal immunity. In the present case, the patient seemed to have CAID that might have contributed to the fatality. Thus, her immunity must have been severely weakened. A late intervention, such as an infection source control or a vital-supported therapy, could be fatal for immune-compromised patients.

Generally, CRP and WBC indicate the inflammation induced by an infection. Thus, CRP level monitoring might be beneficial in detecting the infection, especially in cirrhosis patients [15]. However, Silvestre et al. showed that CRP should not be used as marker of infection in septic patients with severe hepatic failure, as the liver cannot produce enough CRP in response to the inflammation [16]. In cirrhosis patients, the blood overflow into spleen destroys blood cells, resulting in pancytopenia [17]. Therefore, in cirrhosis patients with liver failure, CRP and WBC might not reflect the severity of the inflammation, even in a state of severe sepsis. In fact, the WBC count, hemoglobin levels, and platelet count observed after the initial blood test were relatively low (4,300 cells/μL, 9.3 g/dL, and 42,000 cells/μL, respectively) in our patient. We believed that the CNS damage had caused the fever and that the infection was not severe. The timing of antibiotic treatment was delayed by 24 h after initial onset of symptoms. These data suggest that pseudo-values of inflammation could precipitate patient death. Recently, it has been reported that the thyroid marker procalcitonin (PCT) is a trustful biomarker for bacterial infection, with a sensitivity and specificity of 81.5% and 87.3%, respectively. This biomarker could detect bacterial infection in cirrhosis patients after a relatively short period [18]. Although they were not examined in our case, PCT levels should be examined in patients with cirrhosis who develop high fever.

### CRBSI prevention and case consideration

Prevention measures such as hand hygiene and the use of fully sterile barriers should be implemented during catheter insertion; application of chlorhexidine gluconate for skin antisepsis, maintenance of the sterile technique, and removal of catheters when they are not necessary are effective in preventing infections. In our case, the blood and catheter cultures revealed that *Klebsiella pneumoniae*, a gram-negative rod bacterium that is part of the enteric flora and produces endotoxins, caused a severe infection. The central line had been inserted through the left femoral vein in a sterile fashion and was placed for 2 weeks (Fig. 3). Stools could have possibly contaminated the catheter and thus enabled the inhabiting *Klebsiella pneumoniae* to infect the blood stream. This situation could have been managed using two strategies: an early empirical and over-indicative administration of antibiotics or a strict application of CRBSI preventive measures. The single most important prognostic factor in sepsis is timing of antibiotic administration. In our case, there was a delay of nearly 24 h before antibiotic administration was started. Although the differential diagnosis was challenging and the laboratory results were seemingly normal, infection should always be considered a reason for fever, especially when further signs of systemic inflammatory response syndrome are observed (in this case, hemodynamic compromise and disseminated intravascular coagulation). Moreover, preventive guidelines for CRBSI recommend that CVC lines should be inserted through subclavian or jugular veins; this is not currently obligatory in general practice [19, 20]. We routinely try not to place the catheter at the femoral site. In this case, we had initially inserted the catheter through the right neck and then reinserted it through the left femoral vein because the left neck was infected and not hygienic. Currently, we do not understand whether removal and replacement of CVCs through the left femoral vein is useful in avoiding CRBSI, though it is not recommended to routinely switch the position of or to replace CVCs [19, 21]. Recent randomized control studies also showed no differences in outcome between early CVC removal and watchful waiting [22]. When CRBSI was suspected, fever was mildly high (37.5 °C) (Fig. 2a) and other infections, such as pneumonia or UTI, were also suspected. In this case, watchful waiting instead of a daily CVC removal could be another option. We cannot conclude that long-term CVC insertion causes CRBSI and death, and whether early replacement or watchful waiting is better to prevent CRBSI.

Additionally, the blood endotoxin level was notably high (428.8 pg/mL) in our case, suggesting a probable endotoxin shock. The presence of endotoxemia in patients with septic shock and positive blood culture are related to higher mortality (39%) than that in patients without endotoxemia (7%) [23]. This implies that endotoxemia with sepsis might have worsened the patient’s condition and indicated a poor prognosis in our case. Further investigations are needed.

### Strategy for CRBSI

Patients with severe stroke have a high probability of developing fatal infections, including CRBSI, which is associated with a mortality rate of approximately 10% [24, 25]. However, a tremendous reduction in CRBSI rates by the application of preventative measures has recently been reported in several studies [26–28]. Theoretically and epidemiologically, it is possible and important to prevent CRBSIs, as emphasized by guidelines [19, 20]. However, it is highly challenging to cure CRBSIs once the infection occurs, especially in patients with accompanying organs failure, as in our case. Thus, these patients have a higher risk of death once they develop severe sepsis, including CRBSI.

### Conclusion

CRBSI patients with liver cirrhosis are very challenging to treat. Clinicians should be aware of the possibility of CRBSI in patients with severe stroke and pay more attention to preventive measures, such as avoiding the insertion of CVCs through femoral veins, particularly in patients with accompanying organ failure, as in our case.

## Abbreviations

BP: blood pressure
CRBSI: Catheter related blood stream infection
CRP: c-reactive protein
CSF: cerebrospinal fluid
CT: computed tomography
ER: emergency room
FDP: fibrin/fibrinogen degradation products
HAIs: Healthcare-associated infections
MCA: middle cerebral artery
SAH: subarachnoid hemorrhage
WBC: white blood cell

## References

[1] O'Grady NP, Alexander M, Dellinger EP, Gerberding JL, Heard SO, Maki DG, Masur H, McCormick RD, Mermel LA, Pearson ML, Raad II, Randolph A, Weinstein RA (2002). Guidelines for the prevention of intravascular catheter-related infections. Centers for Disease Control and Prevention. MMWR Recomm Rep.

[2] Siempos II, Kopterides P, Tsangaris I, Dimopoulou I, Armaganidis AE (2009). Impact of catheter-related bloodstream infections on the mortality of critically ill patients: a meta-analysis. Crit Care Med.

[3] Berenholtz SM, Pronovost PJ, Lipsett PA, Hobson D, Earsing K, Farley JE, Milanovich S, Garrett Mayer E, Winters BD, Rubin HR, Dorman T, Perl TM (2004). Eliminating catheter-related bloodstream infections in the intensive care unit. Crit Care Med.

[4] Kamel H, Iadecola C (2012). Brain-immune interactions and ischemic stroke: clinical implications. Arch Neurol.

[5] Dettenkofer M, Ebner W, Els T, Babikir R, Lucking C, Pelz K, Rüden H, Daschner F (2001). Surveillance of nosocomial infections in a neurology intensive care unit. J Neurol.

[6] Ortiz R, Lee K (2006). Nosocomial infections in neurocritical care. Curr Neurol Neurosci Rep.

[7] Klehmet J, Harms H, Richter M, Prass K, Volk HD, Dirnagl U, Meisel A, Meisel C (2009). Stroke-induced immunodepression and post-stroke infections: lessons from the preventive antibacterial therapy in stroke trial. Neuroscience.

[8] Christou L, Pappas G, Falagas ME (2007). Bacterial infection-related morbidity and mortality in cirrhosis. Am J Gastroenterol.

[9] Albillos A, Lario M, Álvarez-Mon M (2014). Cirrhosis-associated immune dysfunction: distinctive features and clinical relevance. J Hepatol.

[10] Wiest R, Lawson M, Geuking M (2014). Pathological bacterial translocation in liver cirrhosis. J Hepatol.

[11] Thalheimer U, Triantos CK, Samonakis DN, Patch D, Burroughs AK (2005). Infection, coagulation, and variceal bleeding in cirrhosis. Gut.

[12] Bonnel AR, Bunchorntavakul C, Reddy KR (2011). Immune dysfunction and infections in patients with cirrhosis. Clin Gastroenterol Hepatol.

[13] Guarner C, Soriano G, Tomas A, Bulbena O, Novella MT, Balanzo J, Vilardell F, Mourelle M, Moncada S (1993). Increased serum nitrite and nitrate levels in patients with cirrhosis: relationship to endotoxemia. Hepatology.

[14] Tandon P, Garcia-Tsao G (2008). Bacterial infections, sepsis, and multiorgan failure in cirrhosis. Semin Liver Dis.

[15] Jalan R, Fernandez J, Wiest R, Schnabl B, Moreau R, Angeli P, Stadlbauer V, Gustot T, Bernardi M, Canton R, Albillos A, Lammert F, Wilmer A, Mookerjee R, Vila J, Garcia-Martinez R, Wendon J, Such J, Cordoba J, Sanyal A, Garcia-Tsao G, Arroyo V, Burroughs A, Ginès P (2014). Bacterial infections in cirrhosis: a position statement based on the EASL special conference 2013. J Hepatol.

[16] Silvestre JP, Coelho LM, Póvoa PM (2010). Impact of fulminant hepatic failure in C-reactive protein?. J Crit Care.

[17] Yongxiang W, Zongfang L, Guowei L, Zongzheng J, Xi C, Tao W (2002). Effects of splenomegaly and splenic macrophage activity in hypersplenism due to cirrhosis. Am J Med.

[18] Li CH, Yang RB, Pang JH, Chang SS, Lin CC, Chen CH, Chen HY, Chiu TF (2011). Procalcitonin as a biomarker for bacterial infections in patients with liver cirrhosis in the emergency department. Acad Emerg Med.

[19] O'Grady NP, Alexander M, Burns LA, Dellinger EP, Garland J, Heard SO, Lipsett PA, Masur H, Mermel LA, Pearson ML, Raad II, Randolph AG, Rupp ME, Saint S, Committee HICPA (2011). Guidelines for the prevention of intravascular catheter-related infections. Am J Infect Control.

[20] Mermel LA, Farr BM, Sherertz RJ, Raad II, O'Grady N, Harris JS, Craven DE, Infectious Diseases Society of America, American College of Critical Care Medicine, Society for Healthcare Epidemiology of America (2001). Guidelines for the management of intravascular catheter-related infections. J Intraven Nurs.

[21] Marschall J, Mermel LA, Fakih M, Hadaway L, Kallen A, O'Grady NP, Pettis AM, Rupp ME, Sandora T, Maragakis LL, Yokoe DS (2014). Strategies to prevent central line-associated bloodstream infections in acute care hospitals: 2014 update. Infect Control Hosp Epidemiol.

[22] Rijnders BJ, Peetermans WE, Verwaest C, Wilmer A, Van Wijngaerden E (2004). Watchful waiting versus immediate catheter removal in ICU patients with suspected catheter-related infection: a randomized trial. Intensive Care Med.

[23] Danner RL, Elin RJ, Hosseini JM, Wesley RA, Reilly JM, Parillo JE (1991). Endotoxemia in human septic shock. Chest.

[24] Zimlichman E, Henderson D, Tamir O, Franz C, Song P, Yamin CK, Keohane C, Denham CR, Bates DW (2013). Health care-associated infections: a meta-analysis of costs and financial impact on the US health care system. JAMA Intern Med.

[25] Umscheid CA, Mitchell MD, Doshi JA, Agarwal R, Williams K, Brennan PJ (2011). Estimating the proportion of healthcare-associated infections that are reasonablypreventable and the related mortality and costs. Infect Control Hosp Epidemiol.

[26] Srinivasan A, Wise M, Bell M, Cardo D, Edwards J, Fridkin S, Jernigan J, Kallen A, McDonald LC, Patel PR, Pollock D, Division of healthcare quality promotion, National Center for Emerging and Zoonotic Infectious Diseases, Centers for Disease Control and Prevention (CDC) (2011). Vital signs: central line-associated blood stream infections–United States, 2001, 2008, and 2009. MMWR Morb Mortal Wkly Rep.

[27] Eggimann P, Harbarth S, Constantin MN, Touveneau S, Chevrolet JC, Pittet D (2000). Impact of a prevention strategy targeted at vascular-access care on incidence of infections acquired inintensive care. Lancet.

[28] Pronovost P, Needham D, Berenholtz S, Sinopoli D, Chu H, Cosgrove S, Sexton B, Hyzy R, Welsh R, Roth G, Bander J, Kepros J, Goeschel C (2006). An intervention to decrease catheter-related bloodstream infections in the ICU. N Engl J Med.

